# Potential Roles of Oral Microbiota in the Pathogenesis of Immunoglobin A Nephropathy

**DOI:** 10.3389/fcimb.2021.652837

**Published:** 2021-04-02

**Authors:** Jia-Wei He, Xu-Jie Zhou, Ping Hou, Yan-Na Wang, Ting Gan, Yang Li, Yang Liu, Li-Jun Liu, Su-Fang Shi, Li Zhu, Ji-Cheng Lv, Hong Zhang

**Affiliations:** Renal Division, Peking University First Hospital, Peking University Institute of Nephrology, Key Laboratory of Renal Disease, Ministry of Health of China, Key Laboratory of Chronic Kidney Disease Prevention and Treatment (Peking University), Ministry of Education, Beijing, China

**Keywords:** IgA nephropathy, oral microbiota, pathogenesis, *Capnocytophaga*, *Rothia*, *Haemophilus*

## Abstract

Disturbance in microbiota affects the mucosal immune response, and it is gradually recognized to be associated with the Immunoglobin A nephropathy (IgAN). This study aims to explore the potential roles of oral microbiota in disease pathogenesis. Saliva samples were collected from 31 patients with IgAN and 30 controls for 16S rRNA gene sequencing. The evenness, diversity, and composition of oral microbiota were analyzed. Moreover, sub-phenotype association analysis was conducted. Phylogenetic Investigation of Communities by Reconstruction of Unobserved States (PICRUSt) based on the Kyoto Encyclopedia of Genes and Genomes (KEGG) database was used to investigate microbiota functions. Compared to healthy controls, microbial diversity tended to decrease in IgAN, and the microbial profiles were remarkably distinguished. The relative abundance of *Capnocytophaga* and *SR1_genera_incertae_sedis* were enriched, whereas 17 genera, such as *Rothia*, were significantly reduced in IgAN. Variable importance in projection scores showed that 12 genera, including *Capnocytophaga*, *Rothia*, and *Haemophilus*, could discriminate between the two groups. In the sub-phenotype correlation analysis, the relative abundance of *Capnocytophaga* and *Haemophilus* was positively associated with levels of proteinuria and serum IgA, respectively. Further metabolic pathway analysis showed 7 predictive functional profiles, including glycosphingolipid biosynthesis, oxidative phosphorylation, and N-glycan biosynthesis were enriched in IgAN. In conclusion, disturbance in oral microbiota was observed to be associated with IgAN and its sub-phenotypes, which may shed novel insights into disease pathogenesis from a microbiome perspective.

## Introduction

Microbial dysbiosis has been shown to be associated with several autoimmune disorders, such as systemic lupus erythematosus, inflammatory bowel disease, rheumatoid arthritis, and multiple sclerosis (Zhang et al., 2015; Cosorich et al., 2017; Vich Vila et al., 2018; Chen et al., 2020). Disturbance in microbiota can affect host immune response, involving maturation and balance of immune cells, the release of various cytokines and chemokines, protection against pathogens, and absorption and metabolism of medicine and nutrients (Belkaid and Hand, 2014).

IgAN is the most common glomerulonephritis in the world, but its pathogenesis is poorly understood. Recent studies highlight the potential roles of microbial dysbiosis in the pathogenesis of IgAN (McCarthy et al., 2011; Kiryluk et al., 2014; Chemouny et al., 2018; He et al., 2020). To date, much of the work focuses on gut-microbiota-associated analysis. However, nasopharynx-associated lymphoid tissue is also an essential component of the host mucosal immune system. Tonsils have been considered one of the primary sources of the poorly O-glycosylated IgA1, which may play a key role in the pathogenesis of IgAN (Horie et al., 2003). Disturbance in microbiota in oral mucosa affects local as well as systematic immune responses. For instance, the dysbiotic microbiome in the local oral cavity determines the periodontitis-associated expansion of Th17 cells (Dutzan et al., 2018). It is also observed that oral pathobionts can translocate to the gut, inducing the migration of Th17 cells, inflammation, and the exacerbation of colitis (Kitamoto et al., 2020). For IgAN, emerging studies begin to investigate the role of oral microbiome profiles (Piccolo et al., 2015; Cao et al., 2018; Luan et al., 2019; Yamaguchi et al., 2021). Changes in the oral microbiome may participate in disease onset and progression. However, there is still a lack of sufficient studies demonstrating the relationship between oral microbiota and the pathogenesis of IgAN.

A better understanding of oral microbiota-immune response dialog may provide novel insights into disease pathogenesis. Thus, in this study, we aim to explore the potential roles of oral microbiota in the pathogenesis of IgAN.

## Materials and Methods

### Study Population

**Figure 1:**
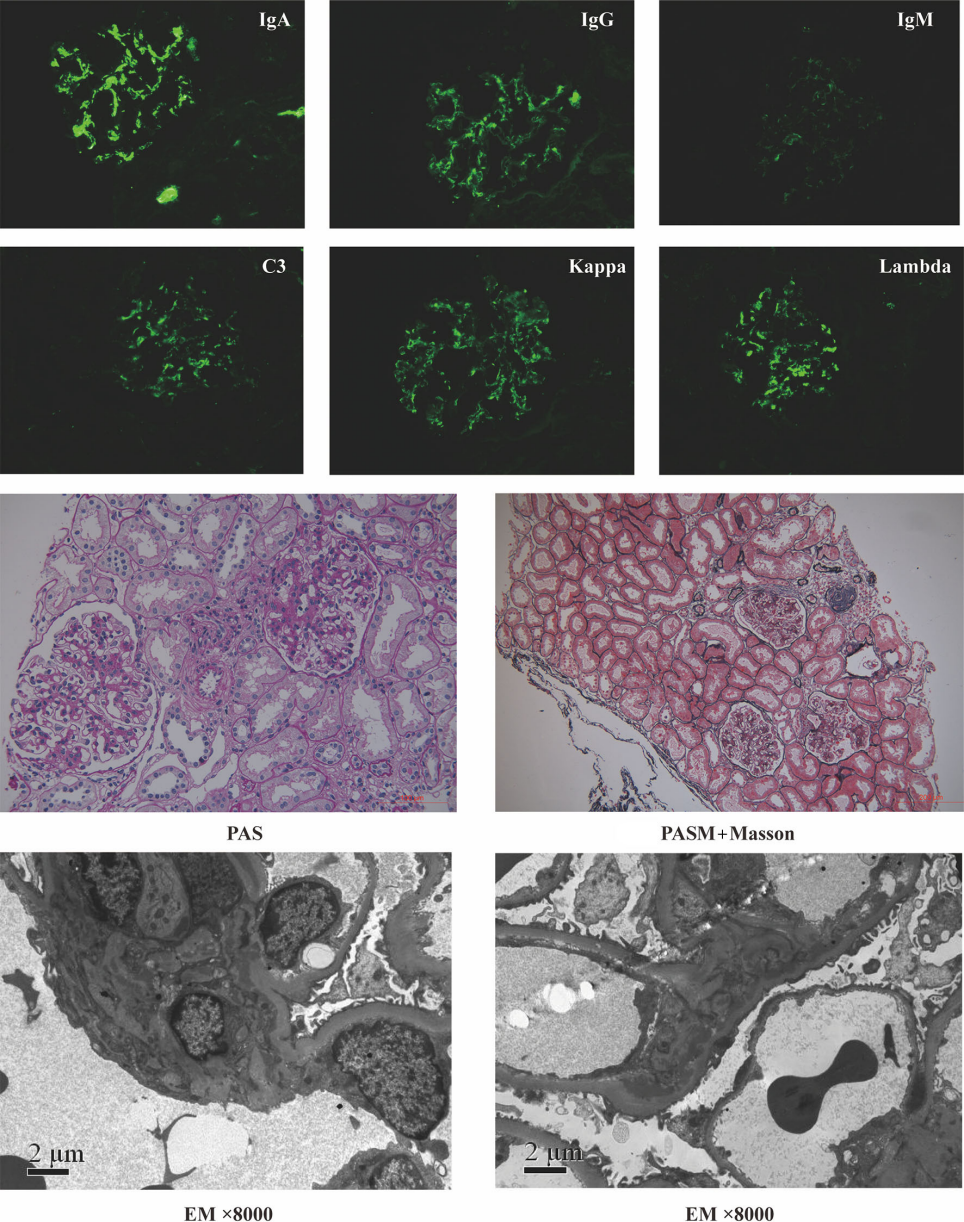
Representative pathology images of patients with IgA nephropathy. PAS, Periodic Acid-Schiff stain; PASM+Masson, periodic acid-silver metheramine+Masson’s trichrome stain; EM, electron microscope.

For microbial analysis, we enrolled participants whose age was >18 y, with Chinese Han ancestry. A total of 61 participants, comprising 31 cases with IgAN and 30 healthy controls (HC), were initially recruited from Peking University First Hospital. The diagnosis of IgAN was confirmed by kidney biopsy with immunofluorescence studies for IgA deposits. Typical pathology images of IgAN were shown in **Figure 1**. Patients with any oral diseases, renal replacement therapies (hemodialysis, peritoneal dialysis, or renal transplantation), secondary IgAN (systematic lupus erythematosus, rheumatic disease, or IgA vasculitis), or type 2 diabetes mellitus were excluded. Ethnically and geographically matched healthy controls were voluntarily recruited. Healthy controls had no oral diseases either. Age, gender, and body mass index were matched between cases and healthy controls.

Those who reported the use of antibiotics, microbial agents, traditional Chinese medicine, glucocorticoid, or other kinds of immunosuppressants within 8 weeks before entry were excluded to minimize the confounding factors. Some supportive medications, such as renin-angiotensin system inhibitors and diuretics, were not controlled.

### 16S rRNA Gene Sequencing

Blood, urine, and saliva samples were collected the day after they were confirmed with IgAN (within one week of kidney biopsy). Patients with fever, cough, or shivers on the day of sample collection were also excluded. All the samples were collected in the hospital and were immediately stored at −80°C until 16S rRNA gene sequencing.

Saliva genomic DNA was extracted using the QIAamp DNA Mini Kit (#51304, QIAGEN). The targeted V3-V4 hypervariable region of the bacterial 16S rRNA genes was amplified by PCR using the primers 341F (5’-CCTACGGGRSGCAGCAG-3’) and 806R (5’-GGACTACVVGGGTATCTAATC-3’). Amplicons were extracted from 2% agarose gels and purified using the AxyPrep DNA Gel Extraction Kit (#AP-GX-50, Axygen Biosciences) and quantified using Qubit2.0 (Invitrogen, MA, USA). After preparing the library, sequencing was performed on a HiSeq platform to generate paired-end reads of 250 bp (Illumina, CA, USA).

### Data Preprocessing

The 16S rRNA gene sequences were processed using USEARCH v.10 and in-house scripts (Edgar, 2010; Zhang et al., 2018). The quality of the paired-end Illumina reads was checked by FastQC v.0.11.5 (Perez-Rubio et al., 2019), and processed in the following steps by VSEARCH: joining of paired-end reads and relabeling of sequencing names; removal of barcodes and primers and filtering of low-quality reads (Q<20); and finding non-redundant reads. Unique reads were denoised into amplicon sequence variants (ASVs)/operational taxonomic units (OTUs). The representative sequences were picked by UPARSEH (Edgar, 2013). The OTU table was generated by USEARCH. The taxonomy of the representative sequences was classified with the RDP classifier (Wang et al., 2007). Analysis of the differential OTU abundance and taxa was performed using the edgeR v3.26.8 package in R v.3.6.1. We subsample (“rarefy”) an OTU table to a fixed number of reads per sample (n=23848) using random subsampling without replacement. Besides, the abnormal observations were filtered if the connectivity was <2.5, using the Weighted Correlation Network Analysis (WGCNA) package (Langfelder and Horvath, 2008).

Diversity analysis was carried out using R (LSD.test function in “agricolae” package) and USEARCH. Alpha diversity was evaluated by the Chao1 diversity index. The significance of differences between the two groups was assessed by permutational multivariate analysis of variance with the Bray–Curtis distance. The number of permutations was 9,999 (vegan package in R). Partial least-squares discriminant analysis (PLS-DA), a supervised learning method, was used to reveal taxonomic changes in different groups. The variable importance in projection (VIP) scores were used to rank the abilities of different taxa to discriminate groups (Rohart et al., 2017). A taxon with a VIP score ≥1 was considered necessary in the group’s discrimination. The comparison of any two groups was conducted by the Wilcoxon rank sum test (two-tailed). The q value represented the False discovery rate-adjusted P-value.

Moreover, we predicted metagenome functional contents from marker gene surveys using the Phylogenetic Investigation of Communities by Reconstruction of Unobserved States (PICRUSt) (Langille et al., 2013). Functional predictions of KEGG Ortholog (KOs) predictions would use the corrected OTU table as input (edge package in R).

### Clinical Variables

Details about the demographics and clinical data, such as age, gender, BMI, blood pressure, urinary sediment microscopy, 24-hour urine protein excretion, serum immunoglobin, complement component 3, and creatinine, were recorded. The estimated glomerular filtration rate (eGFR) was calculated by the modification of diet in renal disease equation. Other serological markers also included levels of serum leukocytes, neutrophils, lymphocytes, monocytes, and high-sensitivity C-reactive protein. All renal biopsies were processed for microscopy (light, electron, immunofluorescence). We scored the renal biopsies according to the Oxford Classification in patients with IgAN (Trimarchi et al., 2017).

### Statistical Analysis

Continuous variables in this study were compared using an unpaired *t* test between groups if the variables were normally distributed; otherwise, a Mann–Whitney U test was performed. Categorical variables were compared using the chi-square test or Fisher’s exact test. Spearman rank correlation coefficient was used for correlation analysis. A two-tailed *P* value of <0.05 was considered statistically significant. The statistical analysis was performed with SPSS 26.0 software (SPSS Inc., USA).

## Results

### General Information of All the Participants

After quality control, we had a total of 60 saliva samples (IgAN=30; HC=30). The age/gender/BMI were matched between cases and controls. Likewise, the proportion of smokers did not differ between the two groups. All the details were shown in **Table 1**.

**Table 1:** Clinical characteristics of all the participants in this study.

Characteristic	IgAN (n=30)	healthy controls (n=30)	*P*-values
Age (years)	38.00 (30.00, 47.00)	34.00 (28.00, 40.00)	0.14
Female (%)	14 (46.67%)	17 (56.67%)	0.44
BMI (kg/m^2^)	23.17 (21.08, 25.43)	22.42 (21.08, 24.03)	0.49
Smoker (%)	5 (16.67%)	4 (13.33%)	1.00
Hypertension (%)	15 (50.00%)		
Hematuria (counts/HPF)	95.40 (32.50, 168.20)		
Proteinuria (g/24h)	2.15 (1.53)		
Serum creatinine (μmol/L)	143.22 (101.90, 186.94)		
Estimated glomerular filtration rate (ml/min/1.73m^2^)	55.32 (32.63)		
Serum IgA (g/L)	3.20 (1.10)		
lEukocytes (10^9^/L)	7.21 (1.66)		
Neutrophils (10^9^/L)	4.78 (1.37)		
Lymphocytes (10^9^/L)	1.60 (1.40, 2.00)		
Monocytes (10^9^/L)	0.50 (0.40, 0.60)		
High-sensitivity C-reactive protein (mg/L)	1.29 (0.59, 2.06)		
Complement C3 (g/L)	0.81 (0.72, 0.93)		
Pathological Oxford classification			
M1	23 (76.67%)		
E1	17 (56.67%)		
S1	21 (70.00%)		
T0	14 (46.67%)		
T1	13 (43.33%)		
T2	3 (10.00%)		
C0	7 (23.33%)		
C1	20 (66.67%)		
C2	3 (10.00%)		

### Profiling of the Oral Microbiota in Individuals With IgAN and Controls

**Figure 2:**
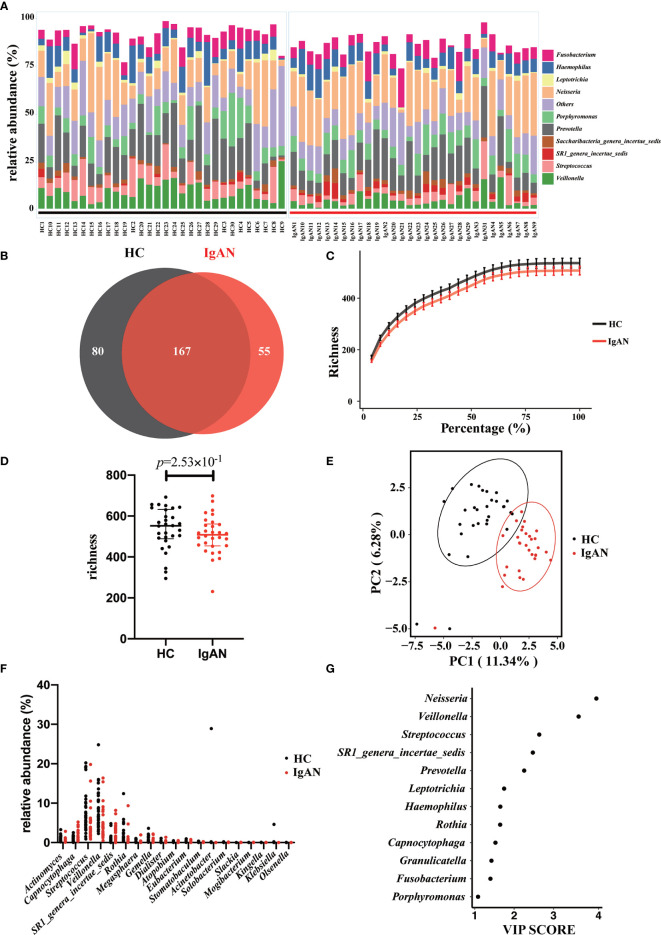
Profiling of the oral microbiota in the present study. **(A)** The bar plot showed the 10 most abundant bacterial genera in patients with IgAN and controls. **(B)** The Venn plot showed the shared and specific OTUs between cases and controls. **(C)** Rarefaction curves were a representation of the species richness for a given number of individual samples. **(D)** As estimated by the richness index, oral microbial diversity tended to reduce in patients with IgAN but without significant difference. **(E)** PLS-DA plot showed a distinct clustering pattern between groups. **(F)** The relative abundance of specific genera with a significant difference among groups (*q*<0.05). **(G)** A taxon with a VIP score ≥1 was considered necessary in the group’s discrimination.

A total of 75 bacterial genera was identified from the salivary 16S rRNA gene sequencing (**Supplementary Table 1**). The ten most abundant bacterial genera based on the 16S sequencing data were displayed in **Figure 2A**. After excluding those unassigned genera, the 10 most predominant genera were *Fusobacterium*/*Haemophilus*/*Leptotrichia*/*Neisseria*/*Porphyromonas*/*Prevotella*/*Saccharibacteria_genera_incertae_sedis*/*SR1_genera_incertae_sedis*/*Streptococcus* and *Veillonella*. The Venn diagram displayed the shared and specific OTUs for the abundant taxa with the mean relative abundance >5×10^-4^. Among 302 OTUs identified using 16S rRNA gene sequencing, 167 OTUs constituted the shared OTUs between cases and controls. Besides, 80 and 55 OTUs belonged to the specific OTUs for controls and patients with IgAN, respectively (**Figure 2B**).

Rarefaction analysis showed that the number of OTUs richness nearly approached saturation in both groups as the percentage of samples increased (**Figure 2C**). Microbial diversity tended to decrease in patients with IgAN. However, at the genus level, no difference in alpha-diversity (richness index) was observed between the two groups (**Figure 2D**). PLS-DA showed a distinct clustering pattern between samples from cases with IgAN and healthy controls (*P*<0.05; **Figure 2E**). We listed the specific genera with a significant difference between the two groups. Two genera, including *Capnocytophaga* and *SR1_genera_incertae_sedis*, were enriched in patients with IgAN, whereas 17 genera including Actinomyces, *Rothia*, and *Dialister* were significantly reduced in patients with IgAN (all *q* values<0.05, **Figure 2F** and **Supplementary Table 2**). Last, the VIP scores were used to reveal which genera contributed significantly to differentiate cases from controls. As shown in **Figure 2G**, 12 genera contributed to the differentiation of the two groups. The top 5 microbes included *Neisseria*/*Veillonella*/*Streptococcus*/*SR1_genera_incertae_sedis* and *Prevotella*.

### Subgroup Analysis of the Oral Microbiota in Individuals With the Same Gender

**Figure 3:**
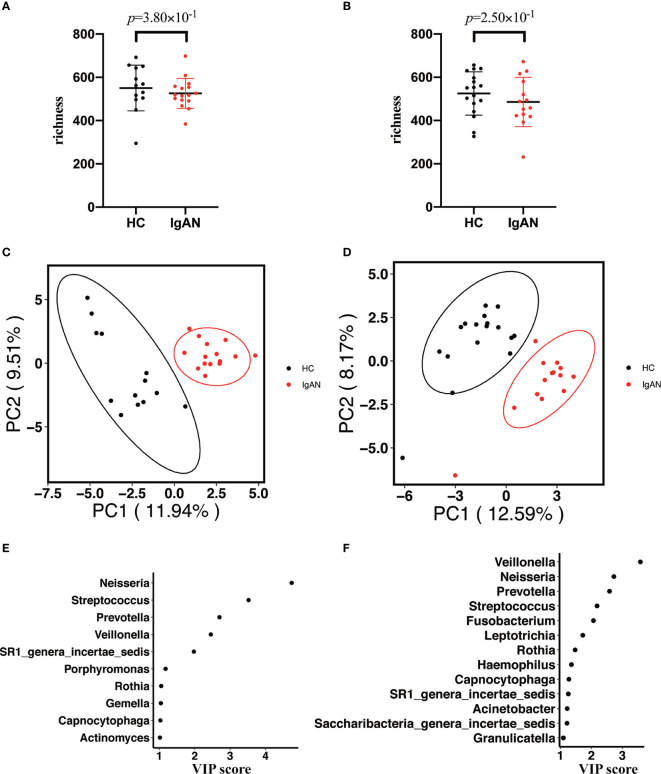
Subgroup analysis of the oral microbiota between cases and controls with the same gender. **(A, B)** The alpha diversity (richness index) between cases and controls in male or female participants (**A** for male participants; **B** for female participants). **(C, D)** PLS-DA plot showed a distinct clustering pattern between groups, regardless of their gender (**C** for male participants; **D** for female participants). **(E, F)** A taxon with a VIP score ≥1 was considered necessary in the group’s discrimination among male and female participants **(E)** for male participants; **(F)** for female participants).

In this study, we enrolled the participants age >18 y. Previous studies indicated that different gender might affect the composition of the microbiota. Thus, a subgroup analysis was performed to study whether the microbial profiles were different between cases and controls with the same gender. Age and BMI were also matched between cases and healthy controls (**Supplementary Table 3**). As shown in **Figure 3**, we didn’t observe that gender significantly affecting oral microbiota composition between IgAN patients and controls. There was no statistical difference in alpha diversity (richness index) between cases and controls with the same gender (male: **Figure 3A**; female **Figure 3B**). Moreover, PLS-DA showed a distinct clustering pattern between patients and healthy controls, regardless of gender (male: **Figure 3C**; female **Figure 3D**). The top four genera that contributed significantly to differentiate cases from controls were the same, including *Neisseria*, *Streptococcus*, *Prevotella*, and *Veillonella*. Moreover, *SR1_genera_incertae_sedis*, *Rothia*, and *Capnocytophaga* also contributed to the group’s differentiation (male: **Figure 3E**; female **Figure 3F**).

### Correlation Analysis Between the Oral Microbiota and Clinical Sub-Phenotypes

**Figure 4:**
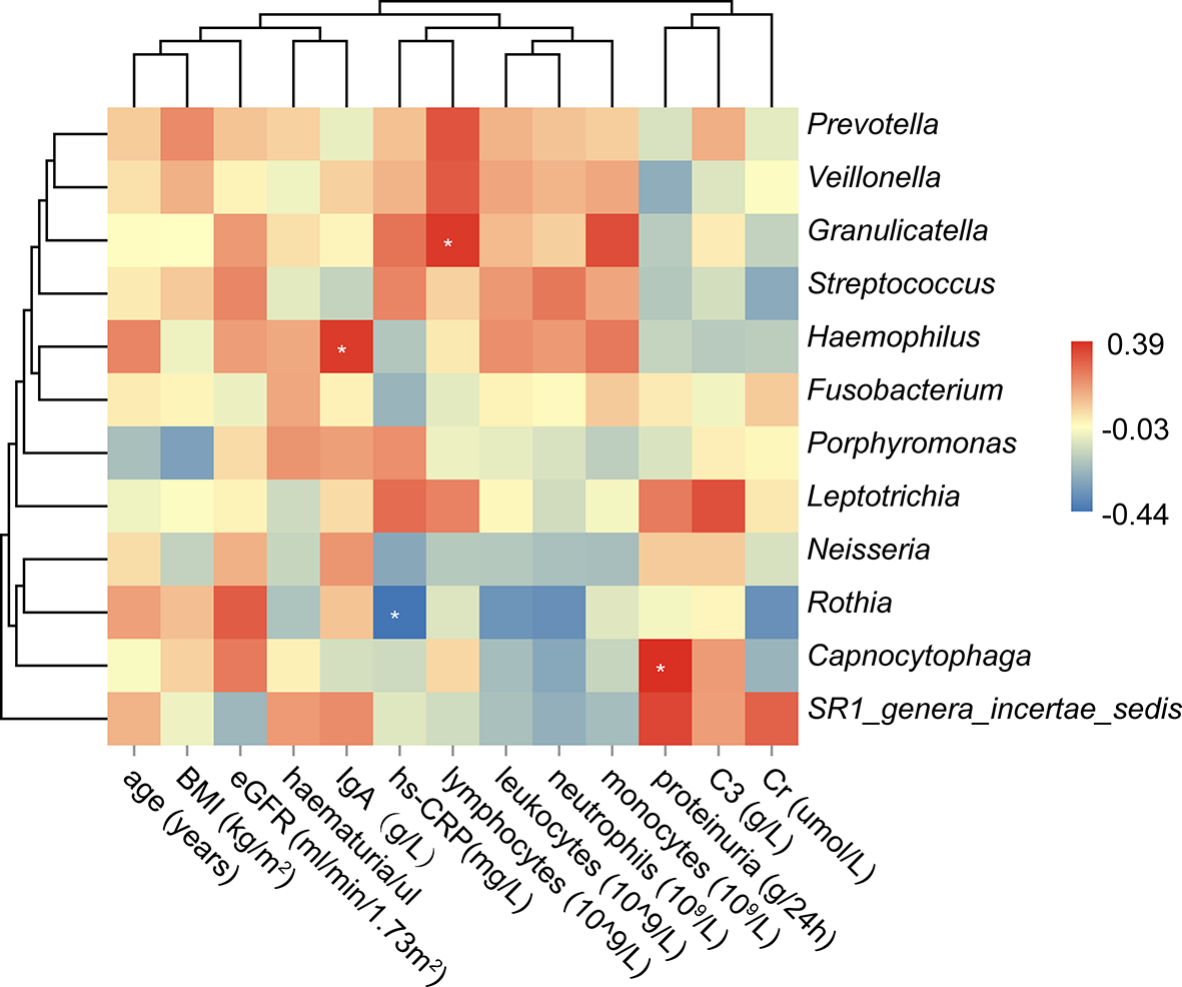
Correlation analysis between the oral microbiota and the clinical sub-phenotypes. The asterisk meant the *P* value<0.05.

We further analyzed the correlations between the oral microbiota and some essential sub-phenotypes (**Figure 4** and **Supplementary Table 4**). It was observed that levels of IgA, hs-CRP, lymphocytes, and proteinuria were associated with the specific microbe. Among all, *Haemophilus* was positively associated with the level of serum IgA (r=0.36, *P*<0.05). *Granulicatella* and *Capnocytophaga* were positively associated with the levels of lymphocytes and proteinuria, respectively (r=0.37, *P*<0.05; r=0.39, *P*<0.05). Instead, *Rothia* was negatively associated with hs-CRP (r=-0.43, *P*<0.05).

### Predictive Functional Profiles That May Be Associated With IgAN

**Figure 5:**
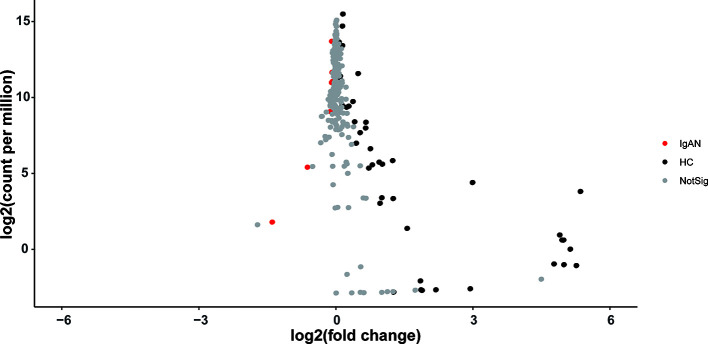
The volcano plot showed distinct KOs between the two groups. Gray points represented those KOs with no significant difference between groups.

The volcano plot showed statistical significance versus the magnitude of change (fold change). As shown in **Figure 5**, only those KEGG Orthologs with *P* value<0.05, FDR<0.1, and logFC thresholds >0 or <0 were colored. In total, 53 KOs with a significant difference were filtered, including 7 KOs (Glycosphingolipid biosynthesis- lacto and neolacto series/peroxisome/oxidative phosphorylation/various types of N-glycan bio-synthesis/streptomycin biosynthesis/MAPK signaling pathway and polyketide sugar unit biosynthesis) were enriched in patients with IgAN. The rest 46 KOs, which were enriched in healthy controls, were shown in **Supplementary Table 5**.

## Discussion

Microbiota interacts with the innate and adaptive immune response. The perturbed microbiota affects the balance of the host immune response (Honda and Littman, 2016; Thaiss et al., 2016). Aberrations in the communication between the host immune system and microbiota may contribute to IgAN.

Our current results showed that perturbations in the oral microbiota might be implicated in IgAN. We conducted the 16S rRNA gene sequencing among age/gender/BMI matched patients and healthy controls. Comparative and association analyses were used to reveal the crucial microbiota and their potential roles in IgAN. Predictive functional profiles provided additional insights into disease etiology and mechanisms. We found that microbial diversity tended to decrease in patients with IgAN but without significant difference. Intriguingly, there was a distinct clustering pattern between cases and controls. Based on the VIP scores, a total of 12 genera were considered necessary to distinguish between cases and controls, including *Capnocytophaga*, *Rothia*, and *Haemophilus*. A subgroup analysis was performed to understand the role of gender on oral microbial profiles. It was observed that gender was not a significant factor impacting oral microbiota between IgAN patients and controls. Results from the alpha diversity analysis, PLS-DA, and VIP index supported the above inference. Moreover, sub-phenotypes association analysis showed that *Capnocytophaga* and *Haemophilus* are positively associated with the levels of proteinuria and serum IgA, respectively. Instead, *Rothia* was negatively associated with the level of hs-CRP. Predictive functional profiles showed that glycosphingolipid biosynthesis, oxidative phosphorylation, polyketide sugar unit biosynthesis, and N-glycan biosynthesis pathways were enriched in IgAN. Analyzing the potential roles of these crucial microbes and those predictive functional profiles can help in further understanding disease pathogenesis.

The decreased *Rothia* and its negative association with hs-CRP might reflect the inflammatory state of the body, which was associated with the production of IgA (Meng et al., 2012). Another study revealed that IgA recognition of the microbiota would be altered during mucosal inflammation status (Palm et al., 2014). In a study about stunted children, several of the highly targeted genera included *Haemophilus* (Huus et al., 2020). *Haemophilus* inhabit the mucous membranes of the upper respiratory tract and mouth. Multiple species of *Haemophilus*, such as *H. influenzae* and *H. ducreyi*, can counter the host immune system and cause human disease. A previous study represented that patients with IgAN had significantly more IgA antibodies against *H. parainfluenzae* than did patients with other glomerular diseases (Suzuki et al., 1994). IgA is a critical mediator of mucosal immune homeostasis. However, excess and poorly O-glycosylated IgA1 seems to play important roles in the pathogenesis of IgAN. Last, *Capnocytophaga* is a commensal genus in the oropharyngeal tract of mammals, which is considered an opportunistic pathogen. In immunocompetent patients, these bacteria are responsible for common kinds of periodontitis (Nonnenmacher et al., 2001). Periodontal-related bacteria affect the host immune response, contributing to IgAN and colitis (Nagasawa et al., 2014; Kitamoto et al., 2020). Consistent with our results, another study confirmed that the relative abundance of *Capnocytophaga* was also increased in chronic periodontitis patients with IgAN (Cao et al., 2018).

Although IgAN is the most common glomerulonephritis globally, the lack of precise therapeutic strategies limits clinical outcomes. Thus, the above results may have significant clinical implications. First, interventions against the specific microbe may relieve the excessive immune responses, reduce the production of poorly O-glycosylated IgA1, and improve clinical outcomes. Some preliminary studies targeting the microbiome in IgAN show promising results (Chemouny et al., 2018). Second, using high-throughput sequencing technologies may contribute to risk stratification. Since the microbial profiles are in a dynamic state, the total relative abundance of some critical microbes (such as *Capnocytophaga*, *Rothia*, and *Haemophilus*) may reflect disease activity. Third, as larger-scale studies continue, using supervised machine learning techniques to analyze the phenotype of microbiota may predict the occurrence of IgAN and assist disease diagnosis. Thus, future studies focusing on the microbiome may shed more light on precision medicine studies.

Through this preliminary study, we shall note some limitations. First, the small sample size precluded analyzing for potential confounders. Microbial profiles will be affected by many factors, such as BMI, gender, alcohol or cigarette consumptions, place of residence, and diet (Vujkovic-Cvijin et al., 2020). Moreover, various kinds of medications have significant impacts on the host microbiome. Except for the antibiotics and glucocorticoid, other drugs, such as metformin, proton pump inhibitors, nonsteroidal anti-inflammatory drugs, atypical antipsychotics, and traditional Chinese medicine, will also affect the composition and the function of our host-microbiome (Forslund et al., 2015; Imhann et al., 2016; Rogers and Aronoff, 2016; Suez et al., 2018; Guo et al., 2020). To avoid host variables confounders and medication impacts, a larger-scale study with IgAN patients and matched healthy controls will be required to clarify the comprehensive microbiome analysis in IgAN. Second, the accuracy observed for reference-based mapping of 16S was much lower. Metagenomic sequencing strategies are recommended for microbiota research. Besides, a multi-omics study, such as blood glycomics, saliva metabolomics, and host genetics in understanding the pathogenic role of oral microbiota in IgAN is warranted. Third, the primary results in our study are based on association analysis. Thus, the validation of the potential roles of these critical microbes in IgAN model animals is required. Understanding how these microbes modulate host immune responses may provide important insights in future intervention studies. Fourth, this study did not enroll patients with other glomerulonephritis in the original study. Thus, we could not know whether these microbial changes were IgAN-specific or shared with chronic kidney disease. More widespread replications and functional studies are still needed in the future. Lastly, the association analyses may not bear multiple corrections. With 12 independent microbial species and 13 clinical variables, a conservative Bonferroni threshold to declare significant association would be 3.21x10^-4^ (0.05/12/13). The indirect effect of oral microbiota in mediating pathogenesis, low statistical power, or sample heterogeneity might account for the marginal significance.

In conclusion, disturbance in oral microbiota was associated with immune response and IgAN. This evidence might provide preliminary clues for future pathogenesis and intervention studies.

## Data Availability Statement

The data presented in the study are deposited in the Genome Sequence Archive (Wang et al., 2017) in National Genomics Data Center (CNCB-NGDC Members and Partners, 2021), China National Center for Bioinformation / Beijing Institute of Genomics, Chinese Academy of Sciences, accession number CRA003794.

## Ethics Statement

The studies involving human participants were reviewed and approved by the Ethics Committee of Peking University First Hospital (Institutional Review Board numbers: 2013 [548] and 2019 [76]). The patients/participants provided their written informed consent to participate in this study.

## Author Contributions

Conceptualization, X-JZ, J-CL, and HZ; methodology, X-JZ, J-WH, PH, and Y-NW; software, J-WH and Y-NW; validation, X-JZ and J-WH; formal analysis, X-JZ and J-WH; investigation, L-JL, S-FS, TG, and YL.; resources, L-JL and S-FS; data curation, X-JZ, J-WH, TG, and YL; writing—original draft preparation, X-JZ and J-WH; writing—review and editing, X-JZ, J-WH, J-CL, and HZ; visualization, X-JZ, J-WH, Y-NW, and TG; supervision, J-CL and HZ; project administration, J-CL and HZ; funding acquisition, X-JZ and J-CL. All authors contributed to the article and approved the submitted version.

## Funding

This research was funded by Beijing Natural Science Foundation, grant number Z190023; National Science Foundation of China, grant numbers 82131430172, 82022010, 81970613, 81925006, 81670649, 82070733; King’s College London-Peking University Health Science Center Joint Institute for Medical Research, grant number 2021; Fok Ying Tung Education Foundation, grant number 171030; Beijing Nova Program Interdisciplinary Cooperation Project, grant number Z191100001119004; Beijing Youth Top-notch Talent Support Program, grant number 2017000021223ZK31; Clinical Medicine Plus X-Young Scholars Project of Peking University, grant number PKU2020LCXQ003; the University of Michigan Health System–Peking University Health Science Center Joint Institute for Translational and Clinical Research, grant number BMU2017JI007; Chinese Academy of Medical Sciences Research Unit, grant number 2019RU023.

## Conflict of Interest

The authors declare that the research was conducted in the absence of any commercial or financial relationships that could be construed as a potential conflict of interest.

## Supplementary Material

Supplementary Table 1 16S rRNA gene data on bacterial taxonomy and relative abundance.

Supplementary Table 2 Specific microbiota with a significant difference between cases and controls.

Supplementary Table 3 Clinical characteristics between cases and controls with the same gender.

Supplementary Table 4 Spearman correlation coefficients and *P* values in the association analysis between oral microbiota and clinical sub-phenotypes.

Supplementary Table 5 Specific KEGG Orthologs with a significant difference between cases and controls.
